# Comparative evaluation of risk management frameworks for U.S. source waters

**DOI:** 10.1002/aws2.1125

**Published:** 2019-02-19

**Authors:** Karen Setty, Robert McConnell, Robert Raucher, Jamie Bartram

**Affiliations:** ^1^ The Water Institute Department of Environmental Sciences and Engineering, Gillings School of Global Public Health, University of North Carolina at Chapel Hill Chapel Hill North Carolina; ^2^ Tampa Bay Water Clearwater Florida; ^3^ Corona Environmental Consulting Louisville Colorado

**Keywords:** drinking water safety, HACCP, risk management, surface water

## Abstract

The U.S. Safe Drinking Water Act required states to develop source water assessment programs identifying existing and potential contamination sources; however, comprehensive risk prioritization and management approaches for surface water supplies have seen limited application. This participatory study assessed which permutation(s) of risk management frameworks and tools might benefit U.S. utilities by combining a literature review with external utility interviews. Qualitative data provided a basis for categorical assignments of goodness of fit with each of 24 framework evaluation criteria across five categories. Weighted integration using stakeholder input provided a relative ranking of applicability, later validated at a decision‐making workshop. Hybridization of the American National Standards Institute/American Water Works Association (ANSI/AWWA G300) source water protection standard and World Health Organization Water Safety Plan guidance was recommended to develop a comprehensive risk management approach for U.S. source waters. Cost–benefit components of other guidance materials were recommended to incorporate financial considerations into risk ranking and mitigation decisions.

## 1 INTRODUCTION

Given growing concerns about environmental pollution and its effects on wildlife and human health, the United States enacted several new environmental regulations in the 1970s, including the Federal Water Pollution Control Act Amendments of 1972. During this period, the 1974 Safe Drinking Water Act (SDWA) sought to protect the quality of drinking water from both surface (e.g., lakes and rivers) and groundwater sources (U.S. Environmental Protection Agency [USEPA], 2017). While groundwater aquifers are sometimes protected by impermeable or filtering geological materials, surface waters tend to receive both point source (e.g., wastewater discharge) and nonpoint source pollution from the upstream watershed (land area that drains into the waterbody). High‐profile spills, disease outbreaks, and drinking water contamination events in the United States serve as reminders that multiple types of risk exist (Allaire, Wu, & Lall, 2018; MacKenzie et al., 1994; Pieper, Tang, & Edwards, 2017; Thomasson et al., 2017). To address vulnerabilities, drinking water suppliers can take measures to prevent the introduction of harmful contaminants, provide resilient services, and protect public health.

The early roots of risk management for public health protection included sanitary inspection in the early 20th century (Wolman, 1921) and the Hazard Analysis and Critical Control Point (HACCP) approach developed in the late 1950s and 1960s to ensure food safety for space missions by the Pillsbury Company, U.S. National Aeronautics and Space Administration, and U.S. Army Laboratories. The HACCP approach, maintained by the U.S. Food and Drug Administration (FDA), has since been widely adopted by the meat, poultry, seafood, dairy, juice, and food service industries. Proactive risk management approaches aimed at ensuring drinking water safety (by considering it a food product) were legislated in other nations, such as Iceland, starting in the mid‐1990s (Gunnarsdottir, Gardarsson, & Bartram, 2012). From 1994 onward, the World Health Organization (WHO) developed tailored risk management guidance for all drinking water suppliers, called a Water Safety Plan (WSP), with global recommendation in the 2004 WHO Guidelines for Drinking‐Water Quality and International Water Association (IWA) Bonn Charter for Safe Drinking Water (IWA, 2004; WHO, 2004).

Drinking water risk management programs have since been implemented in more than 90 countries, including Canada, Australia, and the United Kingdom (WHO & IWA, 2017). As of 2017, policies related to drinking water risk management were in place in at least 46 countries, while 23 countries had policies under development and others reported using them voluntarily (WHO & IWA, 2017). These programs seek to ensure process controls and supply‐chain reliability from source water to the point of consumption (Bartram et al., 2009). They encourage tailoring the risk monitoring and management approaches to each individual water system in addition to the application of national water quality rules across all systems (Baum, Amjad, Luh, & Bartram, 2015). In recent years, several positive outcomes have been associated with proactive drinking water risk management programs, from financial to operational to public health benefits (Gunnarsdottir, Gardarsson, & Bartram, 2012; Gunnarsdottir, Gardarsson, Elliott, et al., 2012; Kot, Castleden, & Gagnon, 2015; Kumpel et al., 2018; Loret et al., 2016; Setty et al., 2017, 2018; String & Lantagne, 2016).

Risk management frameworks and tools used in the U.S. food industry and by drinking water suppliers abroad could benefit drinking water utilities seeking to actively manage source water risks within the United States (Baum, Bartram, & Hrudey, 2016; Havelaar, 1994; Spagnuolo & Cristiani, 2017). Still, drinking water risk management programs have seen limited application in a U.S. context, which is strongly influenced by national regulatory mandates and professional association guidance (Amjad, Luh, Baum, & Bartram, 2016). Since the 1970s, U.S. water quality regulations have continued to rely heavily on reactive compliance monitoring for a nationally standardized list of priority contaminants (Institute of Medicine, 2004). Practical differences exist between U.S. drinking water regulations and risk‐based approaches regarding team procedures and training, internal risk assessment and prioritization, and management procedures and plans (Baum et al., 2015). The 1996 amendments to the SDWA required states to develop source water assessment programs to identify existing and potential contamination sources to drinking water supplies (USEPA, 2018). While this emphasizes state‐level oversight, effective risk management requires engagement at the utility level. Given a comprehensive list of potential hazards, utilities might lack the tools or resources to prioritize and actively manage risks in collaboration with stakeholders in the watershed. The 2018 America's Water Infrastructure Act (AWIA) includes provisions that create regulatory drivers for linking source water protection, vulnerability assessments, and emergency response planning.

A previous Water Research Foundation (WRF) study to develop a preliminary framework for evaluating source water protection programs (4528) recommended evaluating the feasibility of modifying and adopting risk management techniques, such as HACCP, for source water protection (Sham, Sklenar, & Keefe, 2015). To help bridge the gap between source water protection planning and active risk management, this WRF‐sponsored project (WRF, 2019) sought to develop a risk management framework for U.S. source waters, including a comparative evaluation of potential frameworks. These methods go beyond risk identification to help utilities identify ongoing, active strategies useful for managing risks in real time. Ultimately, the project aims to supply pilot testers and framework developers with guidance on which risk management frameworks and tools might be appropriate for large U.S. utilities sourcing water from mixed‐use watersheds with multiple hazards. Implementation science theory helped to assess the strengths and limitations of available guidance frameworks to determine which might thrive in a variety of U.S. settings. The comparative evaluation characterized goodness of fit between several risk management programs and existing utility needs to enable identification of the risk management program(s) most likely to perform well in pilot testing. Researchers considered both literature sources and unpublished knowledge based on the direct experience of international and domestic program users.

## 2 METHODS

To comparatively evaluate source water risk management frameworks and tools appropriate for pilot testing in the United States, the research team undertook a literature review coupled with external utility interviews and participating utility surveys. The review compared and integrated data within a multi‐indicator evaluation matrix to synthesize findings. Results of the external utility interviews were each included as a single reference in the matrix. The Consolidated Framework for Implementation Research (CFIR) and participating utility survey results helped to identify and refine the draft evaluation criteria for comparing different program options, integrating researcher and participant input with existing implementation theory (Damschroder et al., 2009). Researchers shared preliminary results at an in‐person workshop to enable a participatory group decision‐making exercise. This facilitated validation of the evaluation criteria and selection of appropriate source water risk management frameworks for eventual pilot testing.

Four large U.S. drinking water utilities seeking to move from risk identification to active risk management—namely, Tampa Bay Water, Fairfax Water, Greater Cincinnati Water Works, and Suez Water Delaware—participated in the study. Each utility designated two representatives to respond to formal information requests, with involvement and assistance of other internal colleagues as necessary. At the outset of the project, all utilities provided a response to a written 52‐question survey developed by the research team as a starting point for describing their water systems, offering input on the project and establishing a prepilot baseline for eventual comparison with a postpilot evaluation of risk management program status. The University of North Carolina (UNC) Office of Human Research Ethics (IRB #17‐1995) reviewed the project methods, including the participating utility survey and external utility interview guide. WRF sponsored the project, coordinating the review of interim products by an external Project Advisory Committee (PAC) from the United States and Canada consisting of three members with diverse perspectives and expertise.

To cast a wide net, no potential risk management frameworks were excluded. A total of nine frameworks written in English and either currently in use or with potential applicability to source waters were identified for comparison, alongside existing SDWA guidance:

- American National Standards Institute/American Water Works Association (ANSI/AWWA) G300‐14: Standard for Source Water Protection
- ANSI/AWWA J100‐10: Risk and Resilience Management of Water and Wastewater Systems
- FDA: HACCP
- WHO: WSP
- International Organization for Standardization: Food Safety Management Systems (ISO 22000)
- ISO: Risk Management (ISO 31000:2018)
- Australian Standard/New Zealand Standard (AS/NZS 4360:2004 and ISO 31000:2009)
- Australian Drinking Water Guidelines (ADWG)
- European Commission: Techneau Framework and Methods for Integrated Risk Management in Water Safety Plans

### 2.1 Literature review

**Figure 1:**
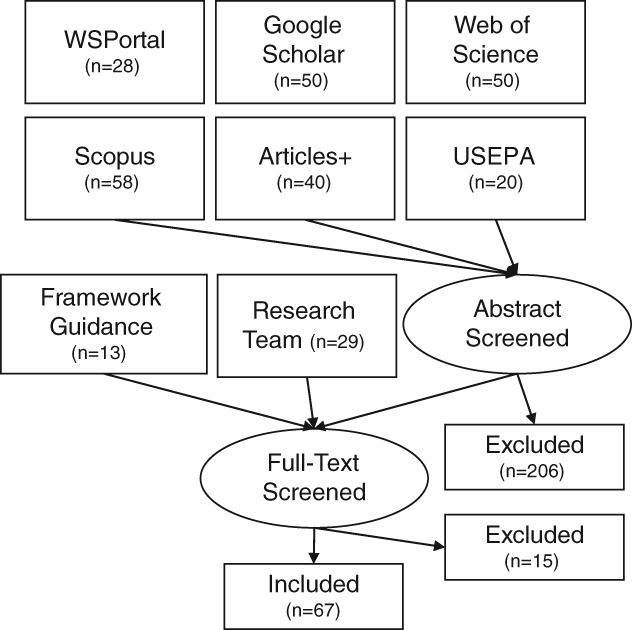
Diagram illustrating literature review sources. USEPA: U.S. Environmental Protection Agency

To review the applicable literature on risk management frameworks and tools used in high‐income countries, researchers gathered (1) risk management framework guidance documents, (2) literature identified via systematic searches of several large databases, and (3) relevant papers submitted by the research team. This included guidance documents, research studies, critical reviews, and case study reports traversing both peer‐reviewed and gray literature. Google Scholar, Web of Science, Scopus, and Articles Plus databases were searched. Boolean operators were used, with search terms including “source, watershed, catchment, drinking, surface, water, utility, risk, safety, plan, prevention, and/or management.” Searches began as narrowly defined and dropped or added terms if too few or too many results were returned, respectively. Generally, the first 50 results were screened by title and abstract. The WHO/IWA Water Safety Portal and USEPA website were also browsed by the topic or geography of interest. Literature inclusion and exclusion criteria are given in Table 1. Full‐text documents from the first round of selected literature were then reviewed and dropped if tangential or narrowly applicable (Figure 1).

**Table 1:** Inclusion and exclusion criteria for source water risk management literature review

Inclusion criteria	Exclusion criteria
Dated 1986 or later (based on date of first SDWA amendment) Relevance to source water Relevance to high‐income nations Inclusion of risk management measures (not just risk identification) Written in English	Single‐contaminant studies Focus on groundwater rather than surface water Focus on small community water systems

*Note.* SDWA: Safe Drinking Water Act.

### 2.2 External utility interviews

To supplement the literature, individual interviews were conducted with utility representatives external to the group of participating utilities. The interview guide (provided as Appendix S1, Supporting Information) covered a general description of the water system; development of the risk management plan, program, or framework; risk identification; risk analysis and evaluation; risk management; and implementation experience. The goal was to interview one practitioner per framework to obtain supplementary information about application in practice. The research team identified approximately 32 potential candidates through networking and interpersonal outreach, of whom 7 ultimately participated in an interview or provided a response to interview questions regarding their utility's experience. Five represented foreign utilities in high‐income countries (Australia, Spain, England, and the Netherlands), and two came from the United States (West Virginia and California), covering six of the evaluated source water risk management frameworks.

### 2.3 Evaluation criteria

The research team drafted evaluation criteria considering the needs expressed during the project proposal stage, organized them into five categories, and revised them based on PAC feedback. Next, the criteria were matched with implementation theory regarding “intervention characteristics” (Damschroder et al., 2009). This required some language adaptation around the level (e.g., practitioner and organization) as CFIR construct definitions more commonly apply to clinical healthcare settings. Finally, criteria were revised and weighted using participating utility survey responses about the relative importance of risk management framework characteristics, assessed both qualitatively and on a Likert scale. Quantitative rankings were used to assign weights (equal to 1, 1.25, or 1.5) to the highest‐priority criteria. Input from the participating utility representatives supported initial weighting, and weights were reassessed following a ranking exercise at the in‐person workshop that included both utility representatives and project advisors.

### 2.4 Data synthesis

An evaluation matrix spreadsheet (available on request) was developed by listing sources (including both literature and interview notes) vertically and evaluation criteria horizontally. To populate the matrix, researchers extracted qualitative information (e.g., quotes, passages, summaries, or presence/absence determinations) upon reviewing the full‐text documents and comparing each source to each evaluation criterion. Cells were tagged using categorical values (*yes* = 2, *maybe* = 1, *no/not applicable* = 0) depending on whether the framework or guidance satisfied or addressed the criterion. This scoring enabled a quantitative sum for relative ranking of the 10 frameworks evaluated. It produced the total number of criteria addressed (with a “yes” answer) for each framework. In addition, weighting was applied to the highest‐priority criteria to produce a summative score for each framework considering all criteria, including “maybe” answers.

The quantitative summary considered framework guidance materials and external interviews but not the additional scientific and gray literature. These supplemental sources often focused on narrower aspects of program evaluation, addressed fewer criteria, and were likely less accessible to a hypothetical program user. In the case of a tie between two sources, the higher‐category score was assigned to the framework. If more than two sources were available, the most prevalent category score was assigned. The additional literature was instead used to develop topic‐specific insight and to create a list of tools (provided as Appendix S1) for addressing specific risk management steps or hazard types. Risk management steps for which specific guidance was available included documentation, rating categories, risk ranking, online water quality monitoring, and validation. Specific risk categories addressed by the literature included climate, supply and demand, main leaks, land use, pathogens, disinfection, fecal indicators, nonpoint sources (e.g., nutrients, sediment), soil erosion, agriculture, emerging contaminants, and pesticides and herbicides.

Preliminary summary results were shared with 14 participants at a decision‐making workshop held in May 3, 2018, in Chapel Hill, North Carolina (Setty, Heymann, Raucher, McConnell, & Bartram, 2018). The workshop consisted of an introductory set of presentations describing the impetus for the research effort, establishing context, and offering historical perspectives and background information on source water protection and risk management methods. Another presentation summarized the methods and findings from the comparative literature review, describing preliminary research recommendations. Participant questions and observations were encouraged to foster dialogue. The workshop also included an interactive group‐learning activity, with participants breaking into small groups for a role‐playing case example in which they developed a generic risk management plan for a hypothetical utility in the southeastern United States. Later, participants engaged in a decision‐making exercise by prioritizing evaluation criteria and ranking risk management frameworks (1) independently based on individual knowledge and preferences and (2) as a group. This Delphi approach to building consensus helped to triangulate and validate the potential applicability of the criteria and frameworks.

## 3 RESULTS

### 3.1 Evaluation criteria

Based on the comparison of draft evaluation criteria with CFIR intervention characteristics, some revisions were made to incorporate constructs that would potentially enhance program feasibility (Table 2). Importantly, the original focus on long‐term sustainability was revised to include both sustainability and short‐term “trialability,” which might benefit implementers who elect to reverse course after testing the program on a small scale. Participating utility survey results were used to revise and develop weighting for the evaluation criteria, focusing primarily on two questions about what factors would be important in selecting a risk management program: “How important are the following criteria to your utility when selecting a source water protection and management program?” (multiple choice) and “Which other criteria, if any, are important to your utility when selecting a source water protection and management program?” (open ended). On the multiple‐choice question, criteria 1.b, 3.a, and 3.c were considered in the range of very important to extremely important to most utilities (detail in Appendix S1) and were assigned a weight of 1.5 (Table 2).

**Table 2:** Evaluation criteria and relationship to CFIR intervention characteristics

Evaluation criteria	Correspondence to CFIR intervention characteristics
1. Implementation feasibility and cost for utility	
a. Relies on readily available and/or readily obtained data	Adaptability
b. References a strategy for coping with data gaps or uncertainties^a^	Adaptability
c. Relies on user‐friendly and readily available tools or methods	Cost and complexity
d. Relies on modest staff time and available in‐house (or external) expertise	Cost and adaptability
e. Flexible and adaptable to low‐to‐modest budget or utility resources^a^	Cost and adaptability
f. Applicable to broad range of source water or watershed risks	Adaptability
g. Applicable to many different types of utilities and geographical settings	Adaptability
h. Sustainable over the long term *and trialable in the short term*	Trialability
2. Risk identification	
a. Provides examples or list of common hazards	Design quality and packaging
b. Readily demonstrates potential hazards to source water	Relative advantage (technical capabilities)
c. Provides relatively comprehensive coverage and identification of potential hazards	Adaptability and design quality and packaging
d. Integrates local or cultural knowledge	Adaptability and intervention source (legitimacy)
3. Risk characterization	
a. Helps quantify or rank identified risks to source water (e.g., to define priorities based on likelihood and consequences)^a^	Relative advantage (technical capabilities)
b. Uses sound science in quantifying and characterizing type and relative level of risk	Relative advantage (technical capabilities)
c. Considers multiple facets of risk (e.g., economic or financial, regulatory compliance, public health, customer relations, and trust or utility reputation)^a^	Relative advantage (technical capabilities)
4. Risk management	
a. Helps identify possible risk‐mitigating options and strategies	Relative advantage (technical capabilities)
b. Helps evaluate and prioritize risk mitigation strategies or options	Relative advantage (technical capabilities)
c. Incorporates monitoring and evaluation strategies (e.g., *in real time* for critical control points)^a^	Relative advantage (technical capabilities)
d. Offers suggestions for program implementation (e.g., identifies best practices or common pitfalls)	Complexity and design quality and packaging
e. Offers advice for managing risks outside the immediate control of utility	Adaptability
f. Incorporates regular feedback loops or quality improvement cycles	Trialability and evidence strength and quality
5. Clarity and ease of communication	
a. Recommends metrics for measuring progress or demonstrating benefits	Trialability and evidence strength and quality
b. Beneficial outcomes have been previously demonstrated *(e.g., employee satisfaction, water quality, public health)*	Evidence strength and quality
c. Supports clarity in conveying risk‐based information: (1) within the utility; (2) with governing boards, public officials, and regulators; and (3) with watershed stakeholders and the general public	Intervention source (legitimacy), evidence strength and quality, complexity, and design quality and packaging

*Note.* Revisions to final criteria in italics. CFIR: Consolidated Framework for Implementation Research.

a Weighted as higher priority.

The open‐ended question showed greater consensus or frequency of stakeholder values related to criteria 1.e, 3.c, and 4.c, which were also weighted as 1.5, while all other criteria were assigned a weight of 1 (Table 2). Although one utility did not provide a response to the open‐ended question, cost was clearly of overriding importance to three of the four participating utilities (matched to criteria 1.e and 3.c). Detecting spill events was mentioned multiple times by a single utility, demonstrating its strength of influence (matched to criterion 4.c). Based on the language used in the open‐ended responses, some criteria were adjusted to better reflect the priorities of the participating utilities. Criterion 4.c, regarding monitoring and evaluation, did not distinguish between real‐time operational monitoring and long‐term compliance monitoring, so “in real time” was added as an example. Similarly, criterion 5.b, which dealt with demonstrated beneficial outcomes, left out the perception of reliability among internal employees. As employee satisfaction with risk management programs is similarly regarded as important in the literature (Gunnarsdottir, Gardarsson, & Bartram, 2012; Summerill, Smith, Webster, & Pollard, 2010), the criterion was revised to incorporate this example.

After receiving and discussing workshop presentations, 10 of the attendees provided input on evaluation criteria by identifying the five criteria most important to their own utility, and then selecting and sharing five they perceived as most important to all utilities. This differed from the written survey question, which requested a single‐utility consensus response. The group activity also added more raters, including external project advisers and coordinators who worked with multiple utilities. Potentially because of these differences, the evaluation criteria ranked as most important for all utilities differed somewhat from the survey responses. Top‐ranked criteria at the workshop (selected by four or more raters) were as follows:

- 1.g: Applicable to many geographical settings and different types of utilities (*n* = 7).
- 3.a: Helps quantify or rank identified risks to source waters (e.g., to define priorities based on likelihood and consequences) (*n* = 7).
- 3.c: Considers multiple facets of risk (e.g., economic or financial, regulatory, public health, customer relations, and trust or utility reputation) (*n* = 7).
- 2.c: Provides relatively comprehensive coverage or identification of potential hazards (*n* = 6).
- 1.a: Relies on readily available and/or readily obtained data (*n* = 4).
- 5.c: Supports clarity in conveying risk‐based information within the utility, governing boards, public officials, regulators, watershed stakeholders, and the general public or customers. (*n* = 4).

Comparing the two rating methods, criterion “3.a helps quantify or rank identified risks to source waters” and criterion “3.c considers multiple facets of risk” remained a high priority. Moderate priority (selection by 40% of raters) was assigned to criteria 1.a (“relies on readily available, and/or readily obtained data”) and 5.c (“supports clarity in conveying risk‐based information”), which were not previously distinguished from other moderately rated criteria (Appendix S1; rank sum = 16). Higher priority (selection by 60–70% of raters) was given to criteria 1.g (“applicable to many geographic settings and different types of utilities”) and 2.c (“provides relatively comprehensive coverage/identification of potential hazards”). In contrast, criterion 1.b (“references a strategy for coping with data gaps or uncertainties”) declined in importance relative to initial weighting.

New criteria raised by workshop participants as highly relevant to risk management program implementation were as follows:

- Connectivity to a support group or body that allows information exchange, continued development, etc.
- Guidance for personnel/human resource aspects (e.g., champion, committees, stakeholder groups, governing boards)

### 3.2 Framework comparison

Of the 67 documents identified for the literature review, 13 were guidance documents, 36 were peer‐reviewed literature sources, and 18 were gray literature sources (Table 3). In most cases, we considered one central framework guidance document per framework, although we attempted to access supporting guidance where possible (e.g., WSP manuals specific to surface water supplies, climate resilience, and auditing). Most of the supplementary literature (*n* = 19) was not particular to a single framework, and some sources (*n* = 4) applied to more than one framework. The majority of the peer‐reviewed literature discussed WSPs (*n* = 20), while most gray literature sources were available for HACCP (*n* = 4). Three interviewees used more than one guidance source, and ISO 22000 was most common (*n* = 3).

**Table 3:** Summary of reviewed literature and interviews by framework

Framework	Guidance documents	Peer‐reviewed literature	Gray literature	Interviews
SDWA^a^	1	0	0	0
ANSI/AWWA G300	1	0	1	1
ANSI/AWWA J100	1	0	1	0
FDA HACCP	1	2	4	1
WHO WSP	4	20	0	2
ISO 22000	1	3	1	3
ISO 31000:2018	1	0	0	0
AS/NZS 4360:2004 and ISO 31000:2009	1	0	1	0
ADWG	1	3	1	2
Techneau	1	0	0	1
Nonspecific	0	10	9	0
Total^b^	13	36	18	7

*Note.* ADWG: Australian Drinking Water Guidelines; ANSI/AWWA: American National Standards Institute/American Water Works Association; AS/NZS: Australian Standard/New Zealand Standard; FDA HACCP: Food and Drug Administration Hazard Analysis and Critical Control Point; ISO: International Organization for Standardization; SDWA: Safe Drinking Water Act; WSP: Water Safety Plan.

a Currently in use (included for comparison).

b Individual totals may sum to more than the total number where documents or interviews cited applicability to more than one framework.

Researchers assigned *yes*, *no*, *maybe*, or *not applicable* scores for each of the 67 literature and seven interview sources across all relevant evaluation criteria (excerpt in Table 4; full list of reviewed literature in Appendix S1; database available on request). Most frameworks, with the exception of WSPs (considering the supporting guidance and interview results), did not address all 24 criteria. Nearly all of the evaluated frameworks met some evaluation criteria, such as incorporating monitoring and evaluation strategies (4.c) and regular feedback loops or quality improvement cycles (4.f). Some criteria were rarely met, such as giving advice for managing risks outside the immediate control of utility (4.e) and metrics for measuring progress or demonstrating benefits (5.a).

**Table 4:** Sample entries from evaluation matrix comparing the primary framework guidance document with 2 of the 24 evaluation criteria

Framework guidance (full reference in supplemental information)	Criterion 1.a: Relies on readily available and/or readily obtained data	Criterion 1.b: References a strategy for coping with data gaps or uncertainties
SDWA	Yes; delineate source protection area and inventory potential contamination sources	No; just includes “known and potential”
ANSI/AWWA G300	Yes; data sources are likely readily available, including “Delineation [of water source geographical area of concern]”; “Water quality and quantity data”; “Contaminant sources, land use, and other threats”; and “Inventory of regulations” (Section 4.2)	Maybe; not discussed as a short‐term issue, although updates are recommended when new data or information becomes available
ANSI/AWWA J100	Maybe; have to characterize assets, vulnerability, and threats	Maybe; quantify consequences based on estimation methods; suggests using midpoints of ranges
HACCP	Maybe; requires pulling multiple health‐research sources: “Considerations of severity (e.g., impact of sequelae, and magnitude and duration of illness or injury)”; “...likely occurrence is usually based upon a combination of experience, epidemiological data, and information in the technical literature”; “The critical limits and criteria for food safety may be derived from sources such as regulatory standards and guidelines, literature surveys, experimental results, and experts.”	No; not apparent
WSP^a^	Yes; requires detailed description including 13 potential data sources. At a minimum, outputs should include (1) description and flow diagram of the system, (2) understanding of current water quality, and (3) identification of users and uses of the water. Must also involve “site visits to confirm the knowledge, information and schematics available to the utility.”	Maybe; names this as a challenge and gives example case studies but does not directly reference a single strategy
ISO 22000	Maybe; rather specific to food production; information requirements include raw materials, ingredients and product‐contact materials, characteristics of end products	No; these issues may challenge the certification effort
ISO 31000:2018	Maybe; gives very brief guidance on what to consider but data sources not specified	Maybe; engagement and awareness of stakeholders “enables organizations to explicitly address uncertainty in decision‐making, while also ensuring that any new or subsequent uncertainty can be taken into account as it arises.”
AS/NZS 4360:2004 and ISO 31000:2009	Yes; characterizes organizational context	Maybe; claims to explicitly take account of uncertainty
ADWG	Yes; for example, assemble historical data from source waters, treatment plants and finished water including exceedances and trend analysis; includes fact sheets with guideline values	Yes; “uncertainty due to lack of knowledge can be reduced through better measurement and research”
Techneau	Maybe; most options in Table 7 require high data availability	Yes; four options for low‐to‐medium data availability presented in Table 7

*Note.* ADWG: Australian Drinking Water Guidelines; ANSI/AWWA: American National Standards Institute/American Water Works Association; AS/NZS: Australian Standard/New Zealand Standard; HACCP: Hazard Analysis and Critical Control Point; ISO: International Organization for Standardization; SDWA: Safe Drinking Water Act; WSP: Water Safety Plan.

a Entry shown for primary guidance manual (Bartram et al., 2009).

When pooled for comparison, the categorical assignment from the written sources and external utility interviews did not always match, as might be expected given the modular presentation of information and differences between guidance and adaptation in practice. In nearly all cases, different sources were within reasonable agreement (e.g., *yes* and *maybe* or *no* and *maybe*). Benefit of the doubt was given to the majority or higher score in case of a tie as described in the methods.

**Figure 2:**
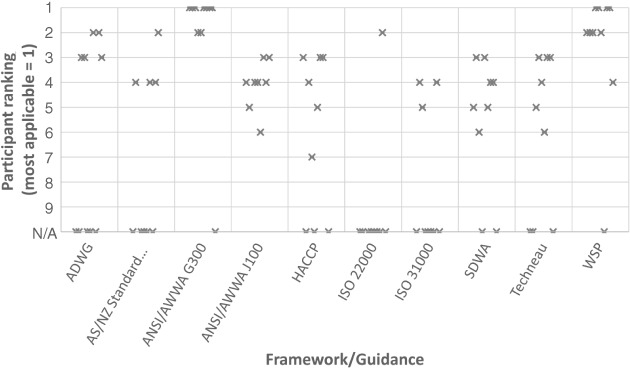
Summary of participant rankings of risk management framework applicability for surface waters in the United States (highest ranking = 1; *not applicable* = N/A). ADWG: Australian Drinking Water Guidelines; ANSI: American National Standards Institute; AS/NZS: Australian Standard/New Zealand Standard; AWWA: American Water Works Association; HACCP: Hazard Analysis and Critical Control Point; ISO: International Organization for Standardization; SDWA: Safe Drinking Water Act; WSP: Water Safety Plan

Based on the data synthesis, a summary of the number of criteria met, weighted score (based on the evaluation criteria and participating utility feedback), description of relative strengths and limitations, and researcher ranking based on context (Table 5) was provided to workshop participants in advance. At the workshop, participants had an opportunity to produce their own ranking (replacing the last two columns of Table 5) individually and shared these rankings with the group as a basis for discussion (Figure 2). They also received a list of tools identified via the literature review for addressing specific risk management steps or hazard categories (provided as Appendix S1).

**Table 5:** Risk management framework applicability for surface waters in the United States, summarizing scores assigned to included framework guidance documents and external utility interviews for 24 evaluation criteria (*yes* = 2, *maybe* = 1, *no/not applicable* = 0; weights = 1 or 1.5)

Framework	No. criteria met	Weighted score	Relative strengths	Relative limitations	Recommended rank based on context^a^	
WSP	24	53	Recommended for worldwide application; extent and nature of implementation largely flexible; many supporting documents (including free guidance on audits)	Primary focus is on protecting human health; less focus on integrating financial considerations; few United States–specific case examples	2	Recommended as a comprehensive risk management framework
Techneau	17	46	Many supporting documents: “structure and toolbox”; spectrum of quantitative and qualitative tool options; intended to improve on WSPs (e.g., by explicitly including risk acceptance/tolerability assessment)	Guidance provision no longer active (website insecure); still somewhat reactive (focused on early warning/response)	3	Recommended for those who would like to explore more advanced, technical options
ANSI/AWWA G300	17	43	United States–centric; respected professional authority; excellent example of watershed outreach in U.S. context; supporting operational guide	Focused on documentation; few case examples provided; requires purchase	1	Recommended as entry point for watershed risk management
ADWG	14	42.5	Comprehensive; user‐friendly; applies elements of HACCP, ISO 9001, and AS/NZS 4360:2004 to drinking water supply	More than 1,000 pages (may be high barrier to entry); developed for Australian context; somewhat focused on water quality	3	Recommended as an example for integrating financial considerations
ISO 22000	15	39.5	Internationally recognized; includes third‐party certification	Tailored to food safety and some analogies (e.g., pest control); may not apply to drinking water facilities; some instinctive rejection of food chain connotation (Deere & Davison, 2008); requires purchase	4	Recommended add‐on only if international recognition is important to utility
ISO 31000	13	37.5	Suggests it can be customized to any organization, sector, or context; simple; gives advice for organizational culture	Requires purchase; cannot be used for certification purposes; brief; not specific to water	—	Not recommended as entry point for watershed risk management
AS/NZS 4360 and ISO 31000	11	36	Certification pertinent to organizations of any kind in Australia and New Zealand; contains many definitions and principles	Requires purchase; layout similar to ISO (not user friendly); not particular to water (or food) safety; diagrams and guidance are somewhat theoretical	—	Not recommended as entry point for watershed risk management
HACCP	11	32	Well‐known; widespread use; can use descriptive historical data to plan for future risks	Specific to food production/not tailored to drinking water or catchments; newer water‐specific guidance available	—	Not recommended as entry point for watershed risk management
ANSI/AWWA J100	10	31	United States–centric; developed by experts after 9/11	Limited adoption; seems burdensome and fairly prescriptive; focused on terrorism and natural hazards; requires purchase	—	Not recommended as entry point for watershed risk management
SDWA	9	30	Existing guidance from U.S. regulations; comprehensive six‐step program	Comprehensive plan not carried out widely in practice; level of expected public involvement may be unrealistic	—	Currently in use (included for comparison)

*Note.* ADWG: Australian Drinking Water Guidelines; ANSI/AWWA: American National Standards Institute/American Water Works Association; AS/NZS: Australian Standard/New Zealand Standard; HACCP: Hazard Analysis and Critical Control Point; ISO: International Organization for Standardization; SDWA: Safe Drinking Water Act; WSP: Water Safety Plan.

a The research recommendation interprets information from the included literature and external case studies before workshop validation or pilot testing. Framework applicability will vary depending on site‐specific context and purpose. For example, HACCP‐based tools were recommended for direct potable reuse source characterization by one participating utility (USEPA & CDM Smith, 2017).

Participant feedback (Figure 2) generally validated findings based on the comparative literature review (Table 5). Individual framework rankings by participants indicated that the ANSI/AWWA G300 and WSP frameworks were the most highly ranked (mostly 1 or 2 of 10 frameworks ranked). The ADWG, AS/NZS, and ISO 22000 ranked fairly well (between two and four) among some participants, while others felt they were not as applicable to U.S. source waters. Reasons included a reasonable length and level of detail, as participants believed that guidance should be distilled and accessible with links to supporting information. Participants also perceived risks of “tokenistic” certification efforts and prescriptive processes distracting from the primary purpose of a risk management program, although a positive accolade could conversely enhance recognition and help to drive performance. The ANSI/AWWA J100, HACCP, ISO 31000, SDWA, and Techneau framework guidance ranked moderately (between three and seven), with participants citing both strengths and limitations. In general, United States–based frameworks were perceived as more applicable to a U.S. context, while foreign guidance was perceived as less applicable.

Updated weights from the participatory ranking exercise were applied to the scoring matrix for sensitivity analysis. The standard weight was set to one, the highest ranked criteria were set to 1.5, and the two intermediate criteria (1.a and 5.c) were set to 1.25. Applying revised weights based on the input of workshop participants, the ranking of frameworks largely remained consistent with the participating utility survey input. Minor fractional differences elevated the SDWA over ANSI/AWWA J100 and the ADWG above ANSI/AWWA G300, which initially had very small degrees of separation (Table 5).

Facilitated discussions following the exercises led to emergence of consensus that two frameworks should be hybridized. ANSI/AWWA G300 offered a suitable foundation given its focus on source water protection, widespread availability, and appeal to U.S. utilities. The WSP framework and supporting materials developed by the WHO, recommended for use in all nations but not yet applied in the United States, were particularly well matched with participants' priorities as a model for holistic risk assessment, risk management, and iterative improvement. Tools drawn from other risk management frameworks, especially the ADWG and Techneau, were recommended to enhance the integration of cost considerations into the overarching framework. ANSI/AWWA J100 also provided examples for calculating the costs of particular hazards.

To summarize the workshop discussion (Setty, Heymann, et al., 2018), participants sought a simple yet strong scientific framework backed by a community of peer and professional support. They believed that all components of a holistic framework or plan were crucial to enable the system to function effectively. Clarity around later steps of risk management, after problem identification, was a particular area of concern. Participants noted that initial team engagement was vital, representing both a primary facilitator and beneficial outcome of risk management. Thus, guidance on how to set people (the primary influencers of risk management programs) up for success was wanting. Participants sought methodological guidance for quantifying and prioritizing diverse risks, broadly defined as any factor that could potentially influence intake water quality or quantity. Utilities faced with time and resource limitations hoped to be able to make good decisions about which risks to actively manage, while at the same time cutting back ineffective programming and gathering more information about poorly characterized risks. Participants likewise valued case examples of correct or successful application of the entire risk management framework.

Participants desired additional guidance on monitoring, since a distinct purpose should always precede monitoring activities, in particular with respect to the differences between proactive operational monitoring and reactive compliance monitoring. Monitoring was common to all risk management frameworks, but the type of monitoring ranged widely, spanning monitoring for compliance, operation, performance assessment, statistical modeling, or public health surveillance (Committee to Review the New York City Watershed Management Strategy, 2000). Some discussion centered on monitoring preventive measures (e.g., operator conducts regular visual checks) versus a hazardous event itself (e.g., a color change in source water). Participating utilities perceived diverse implementation challenges, such as employee turnover, misalignment with organizational priorities, difficulty interacting with powerful watershed stakeholders, and coalescing disparate programs under a cohesive risk management vision, framework, and plan.

## 4 DISCUSSION

This study's results showed distinct gaps where criteria important to drinking water utilities could be addressed using risk management guidance. Among others, these centered on the risk prioritization methods and integration of cost considerations. Participating utilities questioned the relative benefits of quantitative versus qualitative risk assessment and prioritization methods. Both approaches have strengths and weaknesses, and most basic guidance recommends a three‐level or five‐level semiquantitative matrix as a starting point (e.g., Bartram et al., 2009). Malzer, Staben, Hein, and Merkel (2010) found that a simplified three‐level evaluation matrix enabled clearer communication and was more practical than a five‐level matrix. Some risk matrices (e.g., Table 3 in the ADWG) explicitly incorporated cost considerations (NHMRC & NRMMC, 2011). Regardless of the specific method, consistency and transparency were essential. The format of guidance also mattered; some sources were overly brief or lengthy, while others were distributed across an unmanageable number of documents. Workshop participants agreed that the best sources were accessible and user‐friendly, with readily available supplementary material.

Based on the study findings, drinking water risk management guidance for the U.S. context should expressly include mechanisms for considering costs and benefits—for example, to compare the most cost‐effective approaches for mitigating risks. Cost and resource limitations are frequently perceived as a barrier to adopting proactive risk management measures for drinking waters, both in the United States and abroad (Amjad et al., 2016; Gunnarsdottir, Gardarsson, & Bartram, 2012; Jetoo, Grover, & Krantzberg, 2015; Loret et al., 2016; Summerill, Smith, et al., 2010). Additional research evidence about the overall financial costs and benefits of risk management interventions for drinking water utilities in high‐income countries would help to support implementation decisions. Currently, most cost–benefit comparisons focus on low‐ and middle‐income countries, which may differ as a result of greater flexibility in monitoring regimes or poorer initial water efficiency (Hasan & Gerber, 2008; Howard, Godfrey, Tibatemwa, & Niwagaba, 2005). Resource limitations should theoretically be less constraining in the United States, yet they widely affect the drinking water sector (Value of Water Campaign, 2017). Funding investment and requirements associated with AWIA Title II “Drinking Water System Improvement” should help water utilities update their resilience assessment and emergency response plans to incorporate all hazards. This regulatory change adds to the need for guidance on approaches to prioritize and manage risks.

As the main risk‐management program expense is usually staff time (Loret et al., 2016), supervisors could set expectations in advance for time investments to help control costs. U.S. source water protection programs were typically implemented by one or two people at a utility or an external consulting firm. Other approaches call for a team, typically involving at least four to five internal staff members, as well as a managerial champion. The low‐and‐slow investment in getting staff members engaged in and trained on risk management may be more palatable relative to the potentially high financial, societal, reputational, environmental, or public health cost of an unexpected incident. Some organizations choose to use integrated management systems, which link all components of risk (including those based in the catchment and business risks) to a utility‐wide risk management plan (Miller, Guice, & Deere, 2009). One external utility used a technical team to identify risks and develop management options, while a separate business team evaluated the range of lower‐cost to higher‐cost risk mitigation strategies and selected the most sensible one.

Participating utilities additionally perceived information gaps and an overwhelming number of potential risks as limiters of risk management activity. Post, Thompson, and McBean (2017) recommended condensing similar hazards into fewer than 30 categories to avoid a tedious review process. The literature and interviewees often cited the precautionary approach in case of doubt, recommending purposefully higher risk ranking, further monitoring, or watchful waiting to assess shifts in risk (Dominguez‐Chicas & Scrimshaw, 2010). Some experienced practitioners developed actionable categories of preconditions that should be addressed to enable active risk management decisions. These included gathering more information about contaminant sources, monitoring source water for unreported compounds, contingency planning, outreach and education, and seeking relevant policies or regulations. In some cases, the informational and risk avoidance value of upgraded monitoring programs might justify additional instrumental and analytical costs.

While recognizing that multiple barriers offer the greatest protection, drinking water safety efforts in the United States have traditionally focused on the treatment plant. It may be more cost‐effective to enhance upstream barriers in the watershed (e.g., by reducing or eliminating contaminant sources) but politically or logistically more difficult (Committee to Review the New York City Watershed Management Strategy, 2000; Gullick, 2014). Efforts to take customer complaints more seriously were widely recognized as an inexpensive starting point for earlier warning of potential contamination problems (Tang, Wu, Miao, Pollard, & Hrudey, 2013) and commonly coincided with efforts to undertake risk management programming (Kumpel et al., 2018; Setty, O'Flaherty, et al., 2018). Regulatory paradigms were viewed as both a driver and a limiter to risk management programs as they both establish and restrict expectations for practice (Gullick, 2014). Interviewees recognized that the legal framework, degree of regulatory authority and support, and ongoing state of communication and trust occasionally limited their risk management options. All four participating utilities agreed that “lack of authority/regulatory support” was a key drawback to their current programs.

Notably, the programs reviewed here and recommended for pilot implementation at participating utilities are of a voluntary nature. High‐profile recognition or endorsement by innovative utilities or trusted professional authorities (e.g., USEPA, AWWA) may weigh heavily on success when scaling up or adapting drinking water safety programs to new settings. Based on initial project outreach, the USEPA is likely to proceed in the short term by issuing guidance on possible voluntary drinking water risk management approaches, such as those evaluated in this project. This might require clarification of how existing U.S. programs and requirements fit within an overarching risk management umbrella. For example, preliminary guidance provided by AWWA regarding the AWIA identifies “cross‐connections” between separate AWWA standards for source water protection, risk and resiliency, security practices, and emergency preparedness. Under a voluntary model, AWWA could also integrate prospective risk management principles into the retrospective reporting requirements for the Partnership for Safe Water, a subscription‐based treatment and distribution system optimization and recognition program.

Over the long term, direct regulatory support would further build capacity for change (Mercer & Bartram, 2011; Ferrero et al., n.d.), although it would ideally adjust requirements rather than adding to existing compliance fatigue (Amjad et al., 2016). A recent review of repeated water quality violations in the United States raised subnational regulations as a potential solution (Allaire et al., 2018). Substituting a site‐specific, rather than one‐size‐fits‐all, approach could result in greater efficiency and cost savings (String & Lantagne, 2016). For example, the USEPA could allow alternative approaches to drinking water risk management to substitute, in part, for compliance monitoring requirements given the precedent of allowing alternate risk models based on quantitative microbial risk assessment for recreational beach water quality monitoring. However, adapting regulations for human consumption may prove more challenging as drinking water represents a non‐optional and more frequent exposure. To avoid stagnation around minimum requirements (Gullick, 2014), the burden of proof would likely fall on the utility to demonstrate how its risk management program meets or exceeds standard compliance monitoring requirements.

### 4.1 Limitations

Research limitations included potential reporting bias (selective information sharing) in the group workshop setting, wherein utility participants may have felt deferential pressure toward experts. Facilitators from the research team noted the group discussion was somewhat imbalanced early on and made explicit efforts to call upon all utility representatives in later sessions. In the preliminary analysis, only participating utilities provided input on criteria weighting, and slightly different perspectives were evident when comparing two criteria‐weighting methods. For example, a strategy for addressing data gaps and uncertainties was more important to utility staff, while external generalizability to different geographical settings, types of utilities, and potential hazards was more important to external experts.

Some frameworks, including HACCP, WSPs, ISO 22000, and the ADWG, were more widely cited in literature sources (Table 3), leading to a more developed body of knowledge. To avoid bias in interpreting framework guidance, an effort was made to consider diverse language, such as “threats” versus “hazards,” that might address the same evaluation criteria. Some criteria (Table 2) contained split definitions that considered more than one construct (1.g, 1.h, 5.a), were similar to other criteria (2.a and 2.c), or were difficult to measure (1.h). While the research team and external advisors were aware of these issues from the outset of the review, it was ultimately decided to retain the original number of criteria rather than further splitting or combining. The study would have benefited from a second rater, which would enable calculation of inter‐rater reliability metrics for these newly developed criteria.

Cost and accessibility of guidance materials played a limiting role in this study in agreement with Loret et al. (2016). Several documentation resources (especially the ANSI/AWWA G300 *Operational Guide* and ISO standards) required purchase, could not legally be reproduced, or did not exist in an electronic format, which restricted accessibility for review and inclusion in the study. The participating utilities similarly communicated a clear need to justify added risk management programming costs, including subscriptions and fees for documentation. ANSI/AWWA J100 was not formally linked with but might be complemented by separate AWWA‐supported standards and manuals such as G430 *Security Practices for Operation and Management,* G440 *Emergency Preparedness Practices,* and M19 *Emergency Planning for Water and Wastewater Utilities.* An operational guide for ANSI/AWWA J100, which may address more evaluation criteria, is similarly under development. Some other guidance materials were extremely lengthy or had many different supporting documents, which might pose a barrier to quick program startup. Such practicalities of access were listed as the relative strengths and limitations of various frameworks (Table 5).

Finally, while the methods made an effort to incorporate undocumented knowledge via interviews, data interpretation was in some ways limited to the clarity of the authors' or interviewees' presentation of the information. Some program characteristics could differ in practice despite ambiguous written presentation. As different practitioners are likely to have different experiences with the same guidance material, the interview results were not intended to be externally generalizable but to supplement the literature. Despite multiple inquiries, we ultimately could not garner participation in interviews regarding ANSI/AWWA J100, AS/NZS ISO 31000:2009, and ISO 31000, which could illustrate how these frameworks were applied in practice. In accordance with the study goals, sparse application or lack of certification program accessibility among water utilities in the United States could limit peer support networks and, as evidenced by the new evaluation criteria proposed at the workshop, would be considered a drawback to the implementation of these programs.

### 4.2 Recommendations

While the program recommendations are internally valid and specific to this project, external generalizability (e.g., to other U.S. utilities) would be enhanced by utility pilot testing and evaluation. Following this research effort, blended step‐by‐step guidance on the risk management process will be developed to support a 6‐month pilot implementation period at the four participating utilities in 2019. Depending on the outcomes, utilities may elect to incorporate some aspects of the risk management approach into their long‐term programming.

Insights from this study were shared with the AWWA Standards Committee as they review and update ANSI/AWWA G300 in 2018–2019. This was an important project outcome as integrating or transcribing international guidance into national‐level legislation or professional guidance helps to adapt the framework to a narrower context (reducing instinctive rejection of concepts), incentivizes adoption, and creates a stronger basis for program sustainability via ongoing information access and peer‐to‐peer learning communities. Utilities acting as early adopters also provide helpful applied evidence to ease the transition of later adopters (Rogers, 2003). More in‐depth or wider‐scale implementation efforts would be the logical next steps, and hybrid study designs could offer insight about the effectiveness of the intervention as well as the implementation process (Curran, Bauer, Mittman, Pyne, & Stetler, 2012).

At an individual utility level, Sham et al. (2015) recommended a U.S. Centers for Disease Control and Prevention process to assess existing source water protection efforts and tailor programming to the utility's particular goals. Most risk management frameworks recommend a phased approach, recognizing that positive steps are preferred over inaction (Bartram et al., 2009). To overcome the initial challenge of starting a proactive risk management program, both external interviewees and literature sources considered communication and framing important. Stakeholder communication should focus on a common desire of protecting the health of employees, residents, and their families (Summerill, Pollard, & Smith, 2010). A neutral, two‐way, regular communication forum (e.g., watershed management group) could help to facilitate active sharing and translation of ideas. Some reviewed U.S. examples also successfully leveraged external resources to supplement the utility's internal investment.

Known facilitators and benefits of risk management programs are likely to translate to a U.S. context as limited research in the United States has matched findings from other high‐income settings (e.g., Kot et al., 2015; Loret et al., 2016). The USEPA's Water Security Initiative Contamination Warning System pilots (USEPA, 2015) reported seven areas of program benefits. Alert responses became faster over time, corresponding to the findings of Setty, O'Flaherty, et al. (2018) in France. The study similarly demonstrated the value of attention to support from senior management and stakeholder engagement, matching the findings of Gunnarsdottir, Gardarsson, and Bartram (2012) and in Iceland. Similar to the participating utility feedback in this study, both studies described the importance of demonstrating to employees at multiple organizational levels how the project benefitted day‐to‐day operations and utility goals (Gunnarsdottir, Gardarsson, & Bartram, 2012; USEPA, 2015).

Research based in Canada, which in many ways parallels the United States' needs, demonstrated factors related to successful project piloting and scale‐up (Kot, Castleden, & Gagnon, 2017) and mechanisms to better integrate drinking water risk management programs into existing water governance structures (Bereskie, Rodriguez, & Sadiq, 2017). While a limited number of utilities have adopted drinking water risk management programs in Canada, particularly in the province of Alberta, they have not been integrated into the national regulatory framework, as in Australia. Thus, this study may identify synergies for drinking water risk management program applications in Canada as well.

A few interviewed external utility practitioners were using multiple risk management programs, such as national guidance, to comply with legislation alongside a voluntary third‐party certification program, or global guidance transcribed into national legislation. This showed that individual utility approaches need not be limited to one risk management program as program alternatives can be complementary. Even given ideal guidance, implementation can vary widely in practice, and an effort must be made to understand when and how adaptations occur to maintain some degree of fidelity to the designers' intentions (Damschroder et al., 2009). Clear documentation of the risk management program was highly recommended by workshop participants in case of personnel turnover. One participating utility faced an unusually high rate of retirement in the year before project initiation, which utility personnel felt strongly limited their ability to participate. This risk of knowledge loss during transitions may become more prevalent due to the rapidly evolving U.S. water industry workforce.

Since the latter half of the 20th century, tools and guidance for risk management have been updated and improved, becoming more user‐friendly and more closely applicable to drinking water rather than food or other systems. In Iceland, for example, pioneering national legislation in 1995 essentially applied food‐based HACCP principles. Their more recent guidance and training efforts, however, have become more closely aligned with the European Union Drinking Water Directive and WHO's WSP approach for water systems. In addition to the ANSI/AWWA standards updates, a WSP manual update is underway to improve user‐friendliness, for example, by including more diverse case examples (Ojomo, 2017).

Future capacity building should include the criteria raised by workshop participants: connectivity among a supportive network or group to allow information exchange, and guidance for the personnel and human resource aspects critical to the success of risk management approaches. Although the scope of this project was limited to large utilities, consideration of risk management frameworks and tools tailored for use at small U.S. utilities will similarly be vital to ensuring resilient water supplies for all.

## 5 CONCLUSIONS

Considering the needs of utility personnel tasked with source water protection, this comparative evaluation recommended combining ANSI/AWWA G300 and WSPs as user‐friendly guidance sources for managing the risks to U.S. surface water sources. Supplementary techniques and tools could incorporate cost–benefit considerations into risk ranking and mitigation decisions along the lines of examples provided by the ADWG, Techneau, or ANSI/AWWA J100 guidance. WHO and other groups have supplemented and spread WSP guidance through active capacity‐building efforts. The ANSI/AWWA G300 Source Water Protection Standards Committee is likewise making efforts to incorporate aspects of widely used risk management approaches to further improve the program for U.S. utilities.

While some risk management programs were country‐specific or required paid consultation, this review catalogued a variety of approaches and tools available to meet individual utilities' needs and resource levels. It highlighted which considerations might be important when scaling up or adapting drinking water safety programs to other high‐income and heavily regulated settings, such as Canada. Although this study focused on surface water sources in the United States, some findings may apply to other scales of risk management (e.g., including water treatment or distribution systems). Renewed attention to risk management may be the best approach to help prevent unexpected contamination and service interruption events, which ultimately have sizeable effects on the economy and public health.

## Supporting information


**Appendix S1**. External utility interview guide and input from participating utility survey with reviewed literature.
**Table S1**. Ranked criteria out of 12 multiple‐choice options based on four participating utility responses to the baseline survey question “How important are the following criteria to your utility when selecting a source water protection and management program?.”
**Table S2**. Ranked criteria based on qualitative coding of three participating utility responses to the open‐ended baseline survey question “Which other criteria, if any, are important to your utility when selecting a source water protection and management program?.”
