# Supplementary material for: Comparative evaluation of risk management frameworks for U.S. source waters
Source: AWWA Water Sci. 2019 Feb 19;1(1):e1125. doi: 10.1002/aws2.1125 (PMC6450437; doi:10.1002/aws2.1125)
Supplement: Supplementary file 1 — Appendix S1. External utility interview guide and input from participating utility survey with reviewed literature. Table S1. Ranked criteria out of 12 multiple‐choice options based on four participating utility responses to the baseline survey question “How important are the following criteria to your utility when selecting a source water protection and management program?.” Table S2. Ranked criteria based on qualitative coding of three participating utility responses to the open‐ended baseline survey question “Which other criteria, if any, are important to your utility when selecting a source water protection and management program?.” [file AWS2-1-na-s001.docx]

## Supplemental Information: External Utility Interview Guide

**Background:** This questionnaire is being administered for a Water Research Foundation-sponsored project called “Evaluation of Risk Management Frameworks and Tools and Their Application for Managing Source Water Risks in the United States.” The research project is being conducted by Corona Environmental Consulting and the Water Institute at The University of North Carolina, Chapel Hill (UNC). We are gathering information on past experiences with source water risk management approaches from a few international drinking water utilities to help us evaluate program options for the U.S. Your participation is voluntary, and the information you share is considered confidential, so your name will not be used in summary reports. Notes from any follow-up communications will only be shared with members of the research team. If you have any questions or concerns, you may contact [contact information redacted].

**General Water System:** The first section is just to get some background information about your utility.

1. Please briefly describe the nature of your utility (e.g., municipal, special district, private, regional, wholesale, retail).
2. How many people are employed by the utility?
3. How many people are served by the utility (e.g., population or service connections)?
4. About how much drinking water do you provide (e.g., annual average demand, seasonal range)?
5. What are the sources of water supply used by the utility?
   1. Are they primarily surface water, groundwater, or groundwater under the influence of surface water?
6. What are the primary land uses in the watershed (e.g., forested, cropland, rangeland, residential [urban, suburban, rural], commercial, industrial)?
7. What are the greatest source water problems or challenges your utility faces?

**Development of Risk Management Plan, Program, or Framework:** The next section is about how your risk management program came about.

1. What was the motivation for implementing a risk management plan, program, or framework?
2. Which risk management framework(s) is/are currently in use by your utility? (e.g., Water Safety Plans, ISO 22000, AWWA G300-14, AWWA J-100-10, HACCP, Techneau, Australian Standard/New Zealand Standard 4360:2004, Australian Drinking Water Guidelines)
3. Which components of your system are covered (e.g., source water, treatment, distribution)?
4. What was the timeline for development and implementation of the plan, program, or framework?
5. What departments or groups at your utility participated in the development of the plan, program, or framework?
   1. Was there a specific person charged with championing the plan, or a committee, or another entity?
   2. How was the program supported by organizational leadership?
6. Which external stakeholders participated in development of the plan, program, or framework?
7. What percentage of time is spent by employees to support the risk management program?

**Risk Identification:** The next section asks about how you identified different risks to your source waters.

1. What approaches, methods, or tools were used to identify source water risks or hazards?
2. What types of source water risks or hazards were considered?
3. Were problematic data gaps or uncertainties encountered during source water risk or hazard identification?
   1. How much of a challenge did this pose?
   2. How were they addressed?
4. How did you balance between existing issues and issues that could potentially strike in the future?

**Risk Analysis and Evaluation:** Next, we will go over how you reviewed and evaluated the identified potential hazards.

1. What approaches, methods, or tools have been used to analyze and evaluate source water risks…
   1. …to assess probability or likelihood of risks?
   2. …to assess the consequences if these risks did occur?
   3. …to summarize and prioritize the results?
2. Were problematic data gaps or uncertainties encountered during source water risk analysis and evaluation?
   1. How much of a challenge did this pose?
   2. How were they addressed?

**Risk Management:** A third important component of risk management is planning proactive or reactive actions that can be taken to mitigate high-priority risks. Let’s bridge that next.

1. How were source water risk management actions planned or identified?
   1. How many risks were considered for active management?
2. What control indicators are used to help manage source water risks at your utility?
   1. What are the limitations of these indicators?
3. How have you monitored and evaluated the effectiveness of ongoing management actions or control measures?
   1. How successful has this evaluation process been?
4. What approaches have been used to address source water risks in areas that are not directly managed by your utility?
   1. Have you developed an outreach program to work with others in your watershed whose activities may contribute to source water protection or contamination?
   2. What is the level of authority/support from a regulatory standpoint?
   3. How well have these approaches worked for your utility?
5. Have source water risk management activities been linked to other financial asset or risk management activities within your utility?
6. How frequently is your risk management plan, program, or framework updated?
   1. What types of events might trigger an update to the plan, program, or framework?

**Implementation Experience:** Finally, we’d like to learn about how this program worked for you over the long run.

1. What has been your overall experience with implementing the plan, program, or framework?
2. Roughly what percentage of the planned management actions have already been implemented?
   1. What specific components of the plan, program, or framework have been partially implemented or not yet implemented?
3. From your perspective, what have been the general outcomes of your risk management plan, program, or framework?
   1. What have been the outcomes specifically for source water risks?
   2. How have these outcomes been evaluated and/or measured?
   3. How long did it take to achieve these outcomes?
   4. Do you have any specific examples of how your risk management plan avoided or mitigated a potential risk?
4. What have been the biggest challenges to ongoing implementation of the plan, program, or framework?
   1. How have these challenges been addressed?
5. What lessons did you learn from this experience generally?
   1. What did you learn specifically about source water risks?
   2. Are there things that you would do differently if you could do it again?

**Closing:** To wrap up, your insight is invaluable as we work to identify and pilot a source water risk management program in the U.S.

1. Is there anything else important for us to know about your experience with this program?

Thanks so much for your help with our research! If we have any further questions, would it be okay for us to follow up with you?

## Supplemental Information: Input from Participating Utility Survey

Table 1. Ranked criteria out of 12 multiple-choice options based on four participating utility responses to the baseline survey question, “How important are the following criteria to your utility when selecting a source water protection and management program?”

| **Rank Sum^1^** | **Multiple-Choice Response** | **Matching Criteria (*weighted as higher priority)** |
| --- | --- | --- |
| 19 | Allows documentation of data gaps | 1.b. References a strategy for coping with data gaps or uncertainties* |
| 18 | Resilience to uncertainties/data gaps | 1.b. References a strategy for coping with data gaps or uncertainties* |
| 17 | Allows economic valuation of risk avoidance activities | 3.c. Considers multiple facets of risk (e.g., economic/financial, regulatory compliance, public health, customer relations and trust/utility reputation)* |
| 17 | Distinguishes which risks should be actively managed | 3.a. Helps quantify or rank identified risks to source water (e.g., to define priorities based on likelihood and consequences)* |
| 16 | Considers land use and other readily available data | 1.a. Relies on readily available, and/or readily obtained data |
| 16 | Enhances risk communication with external parties | 5.c. Supports clarity in conveying risk-based information: (1) within the utility; (2) governing boards, public officials, and regulators; and (3) watershed stakeholders and the general public |
| 16 | Flexibility/adaptability to different levels of resource availability | 1.e. Flexible/adaptable to low-to-modest budget or utility resources |
| 16 | Integrates quantitative data and local knowledge | 3.a. Helps quantify or rank identified risks to source water (e.g., to define priorities based on likelihood and consequences) AND 2.d. Integrates local or cultural knowledge |
| 16 | Provides examples/list of common hazards | 2.a. Provides examples/list of common hazards |
| 16 | Promotes regular feedback/improvement cycles | 4.f. Incorporates regular feedback loops or quality improvement cycles |
| 14 | Flexibility/adaptability to different types of watersheds | 1.f. Applicable to broad range of source water/watershed risks |
| 14 | Offers advice for managing risks outside immediate control of utility | 4.e. Offers advice for managing risks outside immediate control of utility |

^1^Extremely important = 5, very important = 4, moderately important = 3, slightly important = 2, not at all important = 1; rank sum range = 4–20.

Table 2. Ranked criteria based on qualitative coding of three participating utility responses to the open-ended baseline survey question, “Which other criteria, if any, are important to your utility when selecting a source water protection and management program?”

| **Theme** | **Frequency** | **Matching Criteria (*weighted as higher priority)** |
| --- | --- | --- |
| Cost | 3 | 3.c. Considers multiple facets of risk (e.g., economic/financial, regulatory compliance, public health, customer relations and trust/utility reputation) AND 1.e. Flexible/adaptable to low-to-modest budget or utility resources* |
| Event detection, spills, chemical storage | 3 | 4.c. Incorporates monitoring and evaluation strategies (e.g., for critical control points)* |
| Effective | 2 | 5.b. Beneficial outcomes have been previously demonstrated |
| Reliable | 2 | 5.b. Beneficial outcomes have been previously demonstrated |
| Updated | 1 | 4.f. Incorporates regular feedback loops or quality improvement cycles |
| Health risks | 1 | 5.b. Beneficial outcomes have been previously demonstrated |
| Water quality | 1 | 5.b. Beneficial outcomes have been previously demonstrated |
| Limited staff/resources | 1 | 1.e. Flexible/adaptable to low-to-modest budget or utility resources |
| Inform personnel | 1 | 5.c. Supports clarity in conveying risk-based information: (1) within the utility; (2) governing boards, public officials, and regulators; and (3) watershed stakeholders and the general public |
| Long-term sustainability | 1 | 1.h. Sustainable over the long term/Trialable in the short term |
| Comprehensive | 1 | 2.c. Provides relatively comprehensive coverage/identification of potential hazards |
| Scientific backing | 1 | 3.b. Uses sound science in quantifying/characterizing type and relative level of risk |

## Supplemental Information: Reviewed Literature

Alder, S., Prasuhn, V., Liniger, H., Herweg, K., Hurni, H., Candinas, A., & Gujer, H. U. (2015). A high-resolution map of direct and indirect connectivity of erosion risk areas to surface waters in Switzerland—A risk assessment tool for planning and policy-making. *Land Use Policy*, *48*, 236–249. <https://doi.org/10.1016/j.landusepol.2015.06.001>

Almaarofi, H., A. Etemad-Shahidi, & R. Stewart. (2017). Strategic Evaluation Tool for Surface Water Quality Management Remedies in Drinking Water Catchments. *Water*, *9*(10), 738. <https://doi.org/10.3390/w9100738>

American Water Works Association. (2017). *M50 Water Resources Planning, Third Edition*.

Amjad, U. Q., Luh, J., Baum, R., & Bartram, J. (2016). Water safety plans: bridges and barriers to implementation in North Carolina. *Journal of Water and Health*, *14*(5), 816–826. <https://doi.org/10.2166/wh.2016.011>

*Australian Drinking Water Guidelines: Paper 6 National Water Quality Management Strategy*. (2017) (Version 3.4). Canberra: National Health and Medical Research Council, National Resource Management Ministerial Council.

AWWA Standard: AWWA, 2010. Risk Analysis and Management for Critical Asset Protection (RAMCAP®) Standard for Risk and Resilience Management of Water and Wastewater Systems. AWWA, Denver.

AWWA Standard: AWWA, 2014. ANSI/AWWA G300-14: Source Water Protection. AWWA, Denver.

Barry, S. (1999). HACCP-Based Source Water Protection Program for the Southern Alameda Creek Watershed in Alameda and Santa Clara Counties, California. Retrieved from <http://ucanr.edu/sites/BayAreaRangeland/files/253130.pdf>

Bartram, J., Corrales, L., Davison, A., Deere, D., Drury, D., Gordon, B., … Stevens, M. (2009). Water safety plan manual: step-by-step risk management for drinking-water suppliers. World Health Organization.

Baum, R., & Bartram, J. (2017). A systematic literature review of the enabling environment elements to improve implementation of water safety plans in high-income countries. *Journal of Water and Health*, wh2017175. <https://doi.org/10.2166/wh.2017.175>

Baum, R., Amjad, U., Luh, J., & Bartram, J. (2015). An examination of the potential added value of water safety plans to the United States national drinking water legislation. *International Journal of Hygiene and Environmental Health*, *218*(8), 677–685. <https://doi.org/10.1016/j.ijheh.2014.12.004>

Canadian Water and Wastewater Association (CWWA). (2005). Canadian Guidance Document for Managing Drinking Water Systems: A Risk Assessment/Risk Management Approach. Prepared for Health Canada.

Chiueh, P.-T., Shang, W.-T., & Lo, S.-L. (2012). An Integrated Risk Management Model for Source Water Protection Areas. *International Journal of Environmental Research and Public Health*, *9*(12), 3724–3739. <https://doi.org/10.3390/ijerph9103724>

Clackamas River Basin Council. (2013, May). Organic and Sustainable Farming Certification Report. Retrieved from <http://clackamasproviders.org/images/stories/FullFarmCertificationReport.pdf>

Committee to Review the New York City Watershed Management Strategy. (2000). *Watershed Management for Potable Water Supply: Assessing the New York City Strategy*. Washington, DC: National Academies Press. <https://doi.org/10.17226/9677>

Corrales, J., Naja, G. M., Bhat, M. G., & Miralles-Wilhelm, F. (2014). Modeling a phosphorus credit trading program in an agricultural watershed. *Journal of Environmental Management*, *143*, 162–172. <https://doi.org/10.1016/j.jenvman.2014.04.031>

Davies, J.-M., & Mazumder, A. (2003). Health and environmental policy issues in Canada: the role of watershed management in sustaining clean drinking water quality at surface sources. *Journal of Environmental Management*, *68*(3), 273–286. <https://doi.org/10.1016/S0301-4797(03)00070-7>

Deere, D. (2017). *Climate-resilient water safety plans: managing health risks associated with climate variability and change*. World Health Organization.

Deere, D., & Davison, A. (2008). A HACCP approach to manage water safety for large basins. In J. B. Rose & E. A. Dreelin (Eds.), *Effective cross-border monitoring systems for waterborne microbial pathogens: a plan for action* (pp. 129–149). London: IWA Pub.

Dominguez-Chicas, A., & Scrimshaw, M. D. (2010). Hazard and risk assessment for indirect potable reuse schemes: An approach for use in developing Water Safety Plans. *Water Research*, *44*(20), 6115–6123. <https://doi.org/10.1016/j.watres.2010.07.007>

Elzufon, B. (2015). Collaborating for Healthy Watersheds: How the Municipal & Agricultural Sectors are Partnering to Improve Water Quality. AGree, National Association of Clean Water Agencies, U.S. Water Alliance. Retrieved from <https://www2.nacwa.org/images/stories/public/2015-01-30muni_ag_wp.pdf>

European Committee for Standardization. (2005). Food safety management systems - Requirements for any organization in the food chain (ISO 22000:2005).

Gartner, T., DiFrancesco, K., Ozment, S., Huber-Stearns, H., Lichten, N., & Tognetti, S. (2017). Protecting Drinking Water at the Source: Lessons From US Watershed Investment Programs. *Journal-American Water Works Association*, *109*(4), 30–41.

Goss, M., & Richards, C. (2008). Development of a risk-based index for source water protection planning, which supports the reduction of pathogens from agricultural activity entering water resources. *Journal of Environmental Management*, *87*(4), 623–632. <https://doi.org/10.1016/j.jenvman.2006.12.048>

Gunnarsdóttir, M. J., Gardarsson, S. M., & Bartram, J. (2012). Icelandic experience with water safety plans. *Water Science and Technology*, *65*(2), 277–288.

Gunnarsdottir, M. J., Gardarsson, S. M., Elliott, M., Sigmundsdottir, G., & Bartram, J. (2012). Benefits of Water Safety Plans: Microbiology, Compliance, and Public Health. *Environmental Science & Technology*, *46*(14), 7782–7789. <https://doi.org/10.1021/es300372h>

Havelaar, A. H. (1994). Application of HACCP to drinking water supply. *Food Control*, *5*(3), 145–152.

Hrudey, S. E., Hrudey, E. J., & Pollard, S. J. T. (2006). Risk management for assuring safe drinking water. *Environment International*, *32*(8), 948–957. <https://doi.org/10.1016/j.envint.2006.06.004>

ISO. (2018). ISO 31000: Risk management — Guidelines. Geneva: International Standards Organization.

Jetoo, S., Grover, V., & Krantzberg, G. (2015). The Toledo Drinking Water Advisory: Suggested Application of the Water Safety Planning Approach. *Sustainability*, *7*(8), 9787–9808. <https://doi.org/10.3390/su7089787>

Keirle, R., & Hayes, C. (2007). A review of catchment management in the new context of drinking water safety plans. *Water and Environment Journal*, *21*(3), 208–216. <https://doi.org/10.1111/j.1747-6593.2007.00074.x>

Khan, S. J., Deere, D., Leusch, F. D. L., Humpage, A., Jenkins, M., & Cunliffe, D. (2015). Extreme weather events: Should drinking water quality management systems adapt to changing risk profiles? *Water Research*, *85*, 124–136. <https://doi.org/10.1016/j.watres.2015.08.018>

Kot, M., Castleden, H., & Gagnon, G. A. (2015). The human dimension of water safety plans: a critical review of literature and information gaps. *Environmental Reviews*, *23*(1), 24–29. <https://doi.org/10.1139/er-2014-0030>

Lobley, A., Hayes, C., & Cuthill, A. (2010). Application of an aerial catchment monitoring methodology to assist the development of drinking water safety plans: Application of an aerial catchment methodology for DWSPs. *Water and Environment Journal*, *24*(3), 223–227. <https://doi.org/10.1111/j.1747-6593.2009.00181.x>

Lockhart, G., Oswald, W. E., Hubbard, B., Medlin, E., & Gelting, R. J. (2014). Development of indicators for measuring outcomes of water safety plans. *Journal of Water, Sanitation and Hygiene for Development*, *4*(1), 171. <https://doi.org/10.2166/washdev.2013.159>

López-Roldán, R., Rubalcaba, A., Martin-Alonso, J., González, S., Martí, V., & Cortina, J. L. (2016). Assessment of the water chemical quality improvement based on human health risk indexes: Application to a drinking water treatment plant incorporating membrane technologies. *Science of The Total Environment*, *540*, 334–343. <https://doi.org/10.1016/j.scitotenv.2015.04.045>

Loret, J.-F., Blaudin de Thé, C., Martin-Alonso, Jordi, J., Puigdomenech-Serra, C., Kayser, G. L., & Bartram, J. (2016). Assessing the costs and benefits of Water Safety Plans. Water Safety Portal. Retrieved from <http://www.wsportal.org/wp-content/uploads/2016/11/16-10_IWA_Brisbane_Loret-full_paper.pdf>

Lukas, A., Mayr, E., Richard, L., & Perfler, R. (2011). Supporting the Water Safety Plan (WSP) approach with the Failure Experience Improvement System (FEIS). *Water Science and Technology: Water Supply*, *11*(3), 288–296. <https://doi.org/10.2166/ws.2011.049>

Mälzer, H.-J., Staben, N., Hein, A., & Merkel, W. (2010). Identification, assessment, and control of hazards in water supply: Experiences from water safety plan implementations in Germany. *Water Science and Technology*, *61*(5), 1307–1315. <https://doi.org/10.2166/wst.2010.026>

Martel, K., Kirmeyer, G., Hanson, A., Mullenger, J., & Stevens, M. (2006). *Application of HACCP for distribution system protection*. American Water Works Association.

Miller, R., Guice, J., & Deere, D. (2009). *Risk assessment for drinking water sources*. Research report.

Miller, R., Whitehill, B., & Deere, D. (2005). A national approach to risk assessment for drinking water catchments in Australia. *Water Science and Technology: Water Supply*, *5*(2), 123–134.

National Advisory Committee on Microbiological Criteria for Foods. (1997, August 14). HACCP Principles & Application Guidelines. US Food and Drug Administration. Retrieved from <https://www.fda.gov/Food/GuidanceRegulation/HACCP/ucm2006801.htm>

Olson, G., A. Wilczak, M. Boozarpour, A. Degraca, & J. M. Weintraub. (2017). Evaluating and Prioritizing Contaminants of Emerging Concern in Drinking Water. *Journal - American Water Works Association*, *109*(12), 54–63.

Petterson, S. R., & Ashbolt, N. J. (2016). QMRA and water safety management: review of application in drinking water systems. *Journal of Water and Health*, *14*(4), 571–589. <https://doi.org/10.2166/wh.2016.262>

Pollard, S. J., Strutt, J. E., MacGillivray, B. H., Hamilton, P. D., & Hrudey, S. E. (2004). Risk analysis and management in the water utility sector: a review of drivers, tools and techniques. *Process Safety and Environmental Protection*, *82*(6), 453–462.

Pollard, S., R. Bradshaw, D. Tranfield, J. Charrois, N. Cromar, D. Jalba, … R. Lloyd. (2009). Executive Summary: Developing a Risk Management Culture—Mindfulness in the International Water Utility Sector [Project #3184]. Water Research Foundation. Retrieved from <http://www.waterrf.org/Pages/Projects.aspx?PID=3184>

Post, Y., Thompson, E., & McBean, E. (2017). Insights into the challenges of risk characterization using drinking water safety plans. *Canadian Journal of Civil Engineering*, *44*(5), 321–328. <https://doi.org/10.1139/cjce-2016-0573>

Raucher, B. (n.d.). A risk management framework for source water protection.

Reid, D. C., Abramowski, K., Beier, A., Janzen, A., Lok, D., Mack, H., … Vatcher, R. (2014). Implementation of Alberta’s drinking water safety plans. *Water Quality Research Journal of Canada*, *49*(1), 5. <https://doi.org/10.2166/wqrjc.2013.063>

Rosén, L., Hokstad, P., Lindhe, A., Sklet, S., & Røstum, J. (2007, June). Generic Framework and Methods for Integrated Risk Management in Water Safety Plans. TECHNEAU. Retrieved from <https://www.techneau.org/index.php?id=124>

Schijven, J. F., Teunis, P. F. M., Rutjes, S. A., Bouwknegt, M., & de Roda Husman, A. M. (2011). QMRAspot: A tool for Quantitative Microbial Risk Assessment from surface water to potable water. *Water Research*, *45*(17), 5564–5576. <https://doi.org/10.1016/j.watres.2011.08.024>

Setty, K. E., Kayser, G. L., Bowling, M., Enault, J., Loret, J.-F., Serra, C. P., … Bartram, J. (2017). Water quality, compliance, and health outcomes among utilities implementing Water Safety Plans in France and Spain. *International Journal of Hygiene and Environmental Health*, *220*(3), 513–530. <https://doi.org/10.1016/j.ijheh.2017.02.004>

Sokolova, E., Pettersson, T. J. R., Bergstedt, O., & Hermansson, M. (2013). Hydrodynamic modelling of the microbial water quality in a drinking water source as input for risk reduction management. *Journal of Hydrology*, *497*, 15–23. <https://doi.org/10.1016/j.jhydrol.2013.05.044>

Spiesman, A. L., & Speight, V. L. (2014). A Risk-Based Methodology for Contaminant Prioritization. *Journal - American Water Works Association*, *106*, E150–E159. <https://doi.org/10.5942/jawwa.2014.106.0034>

Standards Australia, & Standards New Zealand. (2009). *Risk management - Principles and guidelines*.

String, G., & Lantagne, D. (2016). A systematic review of outcomes and lessons learned from general, rural, and country-specific Water Safety Plan implementations. *Water Science and Technology: Water Supply*, *16*(6), 1580–1594. <https://doi.org/10.2166/ws.2016.073>

Tang, Y., Wu, S., Miao, X., Pollard, S. J. T., & Hrudey, S. E. (2013). Resilience to evolving drinking water contamination risks: a human error prevention perspective. *Journal of Cleaner Production*, *57*, 228–237. <https://doi.org/10.1016/j.jclepro.2013.06.018>

US Environmental Protection Agency. (2002). Consider the Source: A Pocket Guide to Protecting Your Drinking Water. Retrieved from <https://www.epa.gov/sites/production/files/2015-04/documents/drinking-water-pocket-guide.pdf>

US Environmental Protection Agency. (2014). VSAT 6.0: Vulnerability Self-Assessment Tool. Retrieved from <https://www.epa.gov/waterriskassessment/download-page-vulnerability-self-assessment-tool-vsat>

US Environmental Protection Agency. (2015). Summary of Implementation Approaches and Lessons Learned from the Water Security Initiative Contamination Warning System Pilots.

US Environmental Protection Agency. (2016). Climate Ready Water Utilities: Climate Resilience Evaluation and Awareness Tool Version 3.0 Methodology Guide. Retrieved from <https://www.epa.gov/crwu>

US Environmental Protection Agency. (2016). Online Source Water Quality Monitoring for Water Quality Surveillance and Response Systems. Retrieved from <https://www.epa.gov/sites/production/files/2016-09/documents/online_source_water_monitoring_guidance.pdf>

World Health Organization. (2016). Protecting surface water for health: identifying, assessing, and managing drinking-water quality risks in surface-water catchments. Rickert, B., I. Chorus, O. Schmoll (eds.). Retrieved from <http://www.who.int/water_sanitation_health/publications/pswh/en/>

World Health Organization (WHO) and International Water Association (IWA). (2015). A practical guide to auditing water safety plans.

Yamada, H., Ikeda, M., Suzuki, K., Konishi, S., & Oikawa, T. (2010). Implementation of Water Safety Plan Efforts to Improvement of Source Water Quality in Tokyo Waterworks. *Water Practice and Technology*, *5*(3), wpt2010050–wpt2010050. <https://doi.org/10.2166/wpt.2010.050>

Zaidi, A. Z., & deMonsabert, S. M. (2015). Economic total maximum daily load for watershed-based pollutant trading. *Environmental Science and Pollution Research*, *22*(8), 6308–6324. <https://doi.org/10.1007/s11356-014-3867-7>

## Supplemental Information: List of Tools for Source Water Risk Management Steps or Specific Hazard Categories

| **Risk/Category** | **Reference Title** | **Year** | **Author(s)/Publisher(s)** | **Advantages/Implications** | **Limitations** |
| --- | --- | --- | --- | --- | --- |
| Climate | Climate Ready Water Utilities: Climate Resilience Evaluation and Awareness Tool Version 3.0 Methodology Guide | 2016 | US EPA | US-based; user-friendly | Limited to one category of source water hazards; May not be politically viable in all states |
| Supply/demand | M50 Water Resources Planning, Third Edition | 2017 | AWWA | Centers on water quantity/resource planning; Chapter 7 addresses source water protection (based on AWWA G300) | Voluntary nature of source water protection in both the US and Canada has made it highly flexible but also challenging to implement |
| Main leaks/land use | Application of an aerial catchment monitoring methodology to assist the development of drinking water safety plans: Application of an aerial catchment methodology for DWSPs | 2010 | Lobley et al. | Gained more knowledge about land use in catchments by chartering a helicopter and systematically photographing them | Used for groundwater protection, but could be applied to surface waters; topography/altitude/forested land may be an issue |
| Pathogens | QMRAspot: A tool for Quantitative Microbial Risk Assessment from surface water to potable water | 2011 | Schijven et al. | Supplementary option for quantifying microbial pathogen risks; Free to download | Supported by Netherlands government |
| Pathogens/disinfection | QMRA and water safety management: review of application in drinking water systems | 2016 | Petterson and Ashbolt | Reviews many QMRA examples and tools to support planning for pathogen management | Requires integration with other components of risk management (links to WSP shown in Figure 2) |
| Fecal indicators | Hydrodynamic modelling of the microbial water quality in a drinking water source as input for risk reduction management | 2013 | Sokolova et al. | Fate and transport simulated by a three-dimensional hydrodynamic model for risk decision support | Focused on a single indicator/surrogate; Applied to lake source (river application likely differs) |
| Nonpoint sources e.g., nutrients, sediment | Economic total maximum daily load for watershed-based pollutant trading | 2015 | Zaidi and de Monsabert | Example of water quality trading program to reduce pollution in source waters; Has been applied in several US states | Requires prior EPA approval for anything other than nutrients and sediments |
| Soil erosion/sediment | A high-resolution map of direct and indirect connectivity of erosion risk areas to surface waters in Switzerland—A risk assessment tool for planning and policy-making | 2015 | Alder et al. | GIS map routes runoff and calculates connectivity with source waters; Publicly accessible on the web (http://map.geo.admin.ch) | Map is specific to Switzerland, although approach could be reproduced; May be of limited use for individual utilities |
| Nutrients | Modeling a phosphorus credit trading program in an agricultural watershed | 2014 | Corrales et al. | Credit trading program example from Florida (compared a Least-Cost Abatement approach to a Command-and-Control approach) | Did not attempt implementation |
| Agriculture | Collaborating for Healthy Watersheds: How the Municipal & Agricultural Sectors are Partnering to Improve Water Quality | 2018 | Elzufon | Inspiration from nine US-based water/agricultural collaborations; Diverse examples | All focused on agricultural sources of pollution |
| CECs | Evaluating and Prioritizing Contaminants of Emerging Concern in Drinking Water | 2017 | Olson et al. | Provides context/suggestions for including CECs in risk management approach | Relies on national (top-down) rather than site-specific approach |
| Pesticides/herbicides | Organic and Sustainable Farming Certification Report | 2013 | Clackamas River Basin Council | Summarizes currently available organic and sustainable certifications and cost-share programs; also survey about landowner knowledge and attitudes; provides survey instrument (at end of document) | Examples are specific to Oregon; Knowledge of programs was low among landowners; however, interest was higher in learning more |
| Documentation | Implementation of Alberta's drinking water safety plans | 2014 | Reid et al. | Adapted template for documenting WSPs; Offer government-supported training resources; Distinguishes "short-term measures" that can be taken immediately to provide extra protection | Risk examples are specific to Alberta region; Suggests "acts of God" (very unusual set of circumstances) are much more difficult to predict and require lateral thinking to anticipate |
| Rating categories | Canadian Guidance Document for Managing Drinking Water Systems: A Risk Assessment/Risk Management Approach | 2005 | Canadian Water and Wastewater Association (CWWA) | Provides guidance for a step-wise methodology useable by all drinking water system operators; Rating categories and templates in Annex 3 | Canada-based |
| Risk ranking | An Integrated Risk Management Model for Source Water Protection Areas | 2012 | Chiueh et al. | Similar to approach used by Australian interviewee (land-use based) | Emphasis on source water protection (rather than risk management) |
| Risk ranking | Strategic Evaluation Tool for Surface Water Quality Management Remedies in Drinking Water Catchments | 2017 | Almaarofi et al. | Fuzzy Multiple Criteria Decision Analysis (FMCDA) approach within a typical fixed budget constraint; includes supplementary material | Does not indicate which software packages can/should be used, user needs to parameterize model |
| Risk ranking | A Risk-Based Methodology for Contaminant Prioritization | 2014 | Spiesman and Speight | US-based; uses ratio of concentration to criteria level value for occurrence threshold | Limited to contaminants |
| Risk ranking | VSAT 6.0: Vulnerability Self-Assessment Tool | 2014 | US EPA | Free tool to support risk assessment based on J-100; Easy to understand and use | Focused on acute events; limited applicability to long-term source water improvement or stakeholder involvement |
| Online water quality monitoring | Online Source Water Quality Monitoring For Water Quality Surveillance and Response Systems | 2016 | US EPA | Offers technological solution for real-time source water monitoring; US-based examples | May not be achievable for all utilities (e.g., due to cost); May be a separate (standalone) effort |
